# Identification of endogenous reference genes for RT-qPCR analysis in breast cancer and matched adjacent tissues

**DOI:** 10.3389/fonc.2025.1702210

**Published:** 2026-01-20

**Authors:** Yue Meng, Ya-Wen Wang, Zhi-Bao Xu, Zhong-Qi Qiao, Can Liu, Yan-Duo Chen, Yao Xu, Kai Zhang

**Affiliations:** 1 Department of Breast Surgery, General Surgery, Qilu Hospital of Shandong University, Jinan, Shandong, China; 2 Department of General Surgery, Binzhou Second People’s Hospital, Binzhou, Shandong, China; 3 Department of Thyroid and Breast Surgery, Jinan Third People's Hospital, Jinan, Shandong, China; 4 Department of Pediatric Surgery, Central Hospital Affiliated to Shandong First Medical University, Jinan, Shandong, China; 5 State Key Laboratory for Diagnosis and Treatment of Infectious Diseases, National Clinical Research Center for Infectious Diseases, National Medical Center for Infectious Diseases, Collaborative Innovation Center for Diagnosis and Treatment of Infectious Diseases, The First Affiliated Hospital, Zhejiang University School of Medicine, Hangzhou, China

**Keywords:** real-time quantitative PCR (RT-qPCR), endogenous reference genes (RGs), breast cancer, 18S rRNA, TFRC

## Abstract

**Background:**

Real-time quantitative PCR (RT-qPCR), essential for gene expression and biomarker studies, requires stable endogenous reference genes (RGs) for normalization. This study aimed to identify consistently expressed RGs in breast cancer and adjacent tissues to facilitate comparative analyses of breast cancer-related gene expression.

**Material and methods:**

Five candidate RGs (β-actin, 18S rRNA, PUM1, RPLP0, TFRC) were analyzed by RT-qPCR from 30 breast cancer patients. Threshold cycle (Ct) values were evaluated using descriptive statistics, and stability of RGs was assessed using RefFinder, integrating GeNorm, NormFinder, ΔCt, and BestKeeper algorithms.

**Results:**

In cancer tissues, descriptive statistics showed that 18S rRNA was a suitable RG (Ct Range = 3.96; Mean Ct=8.43; SD = 0.84). RefFinder identified TFRC as the most stable RG (GM = 1.19), followed by 18S rRNA (GM = 1.41). In adjacent tissues, TFRC was find with its narrowest Ct range (Ct range = 6.29) and highest stability (GM = 1.00) by RefFinder. However, GeNorm and BestKeeper indicated instability for TFRC (M = 2.364, SD = 4.30), exceeding the stability threshold values of 1.5 and 1, respectively. Adjacent tissues displayed significantly higher Ct values than cancer tissues. TFRC may serve as the suitable RG for detecting gene expression when concerning both breast cancer and adjacent tissues (GM = 1.19), though, GeNorm and BestKeeper indicated its instability (M = 2.364, SD = 4.30).

**Conclusion:**

TFRC and 18S rRNA may be suitable RGs in breast cancer tissues, while all five candidates were not stable in adjacent tissues. Larger studies are needed to confirm these findings.

## Highlights

- TFRC and 18S rRNA are potential endogenous reference genes for breast cancer tissues.
- GeNorm, NormFinder, BestKeeper, and RefFinder were used to evaluate RGs.
- Integrating clinicopathological parameters enhances RGs selection for breast cancer.

## 1 Background

Breast cancer is the leading cause of cancer-related mortality among women, with 670,000 deaths annually. It ranks as the fourth most common cancer-related cause of death globally (1). In China, the incidence of breast cancer is the fourth highest, and it has been rapidly increasing since the 1990s, at a rate twice as fast as the global average, particularly in urban areas (2). Recent evidence also indicates a shift toward younger age at diagnosis, with an increasing proportion of cases occurring in younger women (3).

Traditional tumor characteristics such as tumor size, grade, and lymph node metastasis are insufficient for evaluating the treatment response and prognosis of breast cancer patients. Consequently, many studies have focused on identifying biomarkers for early diagnosis, treatment, and prognosis of breast cancer. Estrogen receptor (ER), progesterone receptor (PR), HER2, and Ki-67 are common markers for guiding adjuvant therapy (4). Standard methods for detecting qualitative biomarkers, such as immunohistochemistry (IHC) and enzyme-linked immunosorbent assay (ELISA), have been proven to be feasible (5). However, these methods are limited by their inability to quantify gene expression and their time-consuming nature. In contrast, RT-qPCR offers the potential to improve the accuracy of early breast cancer diagnosis and prognostic analysis. However, RT-qPCR results can be influenced by variations in initial sample size, RNA integrity, and reverse transcription efficiency (6). Endogenous reference genes (RGs) are genes whose expression remains relatively constant. It is essential to select appropriate RGs under specific experimental conditions to ensure accurate normalization.

Despite the importance of RGs, there is no consensus on the optimal RGs for gene expression studies in breast cancer. Several studies have identified some potential RGs. For instance, an analysis of 12 commonly used RGs across 23 cancer cell lines indicated that 18S rRNA is stable across a broad range of cancer types, while PUM1 was identified as a suitable RG for breast cancer (7). PUM1 has been reported as a reliable RG in both breast cancer and normal breast tissues (8, 9). Combining three RGs (TBP, RPLP0, and PUM1), or using PUM1 alone, is considered a suitable approach for normalizing gene expression in breast cancer and adjacent normal tissues (10). Other studies have suggested that β-actin is widely used as an RG in breast cancer research (11–14). RPLP0 and β-actin have also been shown to be stable RGs in breast cancer (15), remaining reliable even in paraffin-embedded and long-term cryopreserved samples (16).

For RT-qPCR data analysis, appropriate RGs are required for processing Ct values. Several algorithms, including geNorm (17), NormFinder (18), BestKeeper (19), and ΔCt (20), have been developed to assess the stability of RGs. These tools help identify the most suitable RGs under different experimental conditions, cell types, and tissues. The software RefFinder (21) integrates the results of above four algorithms to select the most appropriate RGs. RefFinder has been used in various cancer-related studies to identify suitable RGs, such as screening potential RGs for radiation therapy response in colorectal cancer (22) and identifying appropriate RGs in periprostatic adipose tissue under obesity and prostate cancer (23).

In this study, five commonly used RGs (β-actin, 18S rRNA, PUM1, RPLP0, and TFRC) were evaluated for their stability in breast cancer and matched adjacent tissues. RT-qPCR data were analyzed using the aforementioned algorithms. RefFinder was used to assess the expression stability of these five candidate RGs.

## 2 Materials and methods

### 2.1 Clinical samples

Breast cancer tissues and matched adjacent tissues were collected from thirty breast cancer patients at Qilu Hospital, Shandong University, between September 2017 and May 2018. Tumor tissue was histologically confirmed as malignant. The adjacent tissue was sampled from a macroscopically normal area located ≥2.0 cm from the visible tumor margin and was pathologically verified to be free of tumor cell infiltration (24–26). The tissues were rapidly frozen in liquid nitrogen and subsequently stored at -80 °C. This study was approved by the Ethics Committee of Qilu Hospital, Shandong University. The clinical and histopathological features of the patients are shown in **Supplementary Table S1**.

### 2.2 Selection criteria for RGs

The selection criteria for RGs included: (1) documented use or reported stability in breast cancer or related tissues; (2) representation of different biological categories; and (3) availability of primer pairs that passed preliminary specificity testing. The candidate RGs were pre-screened in four breast cancer cell lines (MDA-MB-231, BT-474, MCF-7, MDA-MB-468) and those showing stable Ct values were advanced to tissue-based stability validation.

### 2.3 RNA extraction and reverse transcription

Total RNA was extracted from the tissue samples using the RNA-easy Isolation Reagent (Vazyme, China). RNA concentration was determined using a spectrophotometer (SMA4000, Merinton, China). RNA was reverse transcribed into complementary DNA (cDNA) using the ProFlex™ PCR System (Applied Biosystems, USA), catalyzed by HiScript III RT SuperMix for qPCR (+gDNA wiper) (Vazyme, China).

### 2.4 Real-time quantitative PCR

The RT-qPCR reaction was performed using ChamQ SYBR qPCR Master Mix (Vazyme, China). The reaction conditions were as follows: initial denaturation for 30 seconds at 95 °C, followed by 40 cycles of denaturation for 10 seconds at 95 °C and annealing for 30 seconds at 60 °C, using the QuantStudio 3 PCR instrument (Applied Biosystems, USA). Each sample was repeated three times.

### 2.5 Data analysis

Statistical analysis was performed by SPSS 19.0 (IBM SPSS Statistics, Armonk, USA) and Graphpad Prism 9.0 (GraphPad Software, San Diego, USA). The normality of the Ct values was assessed using the Shapiro-Wilk test. T-tests or non-parametric tests were employed to examine the differential expression of candidate RGs between breast cancer tissues and matched adjacent tissues. Samples were categorized into subgroups based on clinical and histopathological parameters, and differences in Ct values were evaluated using 2-way ANOVA. P< 0.05 was considered statistically significant.

Five candidate RGs were analyzed using RefFinder, an online tool for the comprehensive evaluation of RGs. RefFinder integrates four widely used algorithms—geNorm, NormFinder, BestKeeper, and ΔCt—to assess the stability of potential RGs (20).

GeNorm is used to evaluate the expression stability of genes across different samples. The Ct values of candidate RGs are converted into M values (average expression stability), with lower M values indicating more stable expression. If the M value exceeds 1.5, the gene is considered unsuitable as an RG. Additionally, the optimal number of RGs is determined by calculating the pairwise variation (Vn/Vn+1). When this value is less than 0.15, the selected number of RGs is considered sufficient for accurate normalization of RT-qPCR data (16). NormFinder ranks RGs based on their stability value (SV), with the gene exhibiting the lowest SV being considered the most stable (17). The ΔCt method ranks candidate RGs based on the average Ct (Avg.Ct) and compares the relative expression of pairs of genes within each sample (19). BestKeeper evaluates the stability of RGs based on the coefficient of variation (CV) and the relative standard deviation (SD), with genes showing an SD greater than 1.0 considered unsuitable (18).

## 3 Result

### 3.1 Expression analysis of the reference genes by Ct value

The five candidate reference genes (β-actin, 18S rRNA, PUM1, RPLP0, and TFRC) were pre-validated in four breast cancer cell lines (**Supplementary Figure S1**). Stable expression of these five candidate reference genes was observed in breast cancer cells, thus they were selected for subsequent tissue-level validation. All reference gene primers exhibited single, specific melting peaks and acceptable amplification efficiencies. After confirming the stability of Ct values, tissue-based stability validation was performed on the stably expressed genes (**Supplementary Figure S2**).

**Figure 1:**
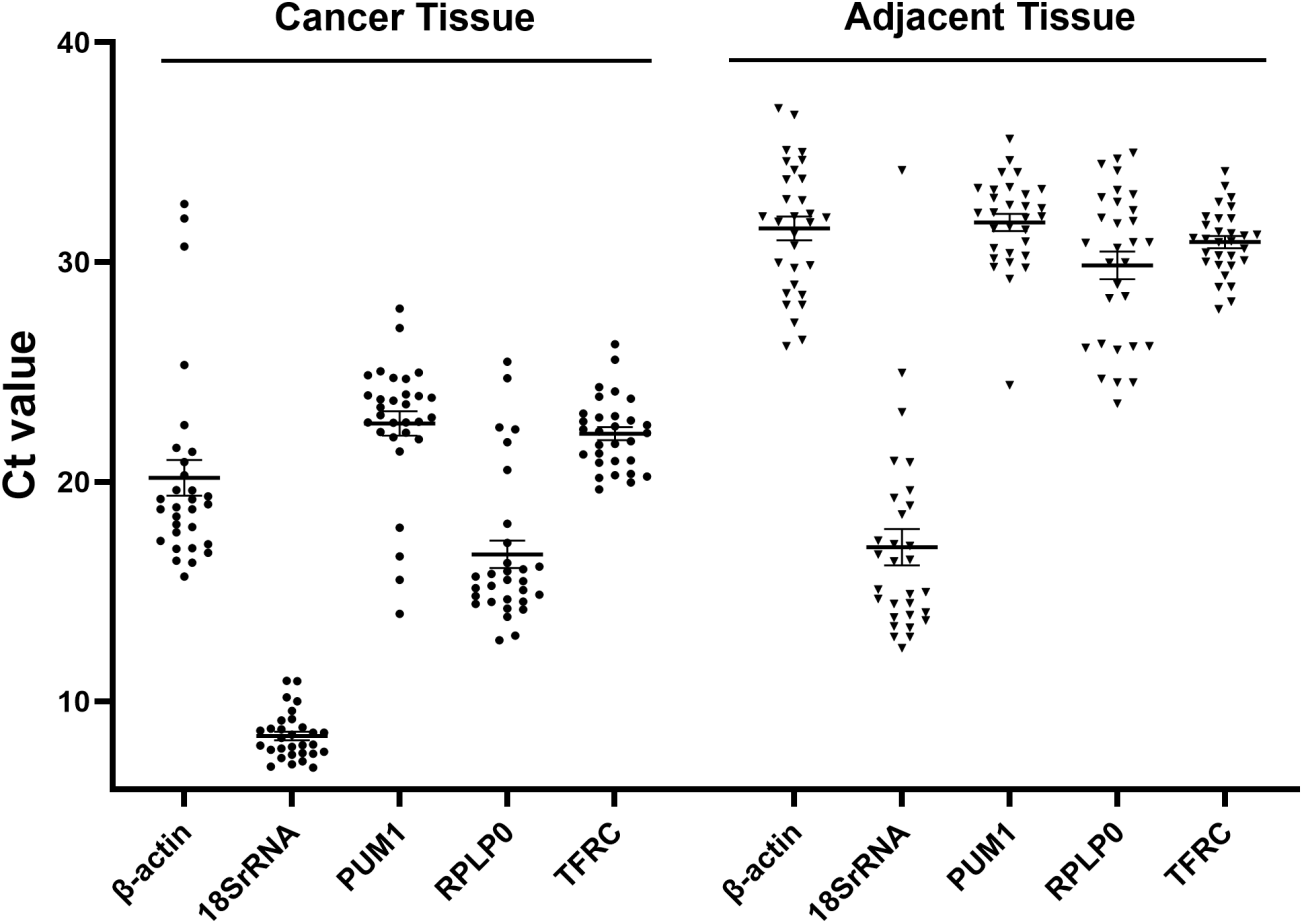
The median and quartile of five candidate RGs in 30 pairs of tissue samples. The line represents the median, and the target value represents the 25th and 75th percentiles.

The Ct values of five candidate RGs (β-actin, 18S rRNA, PUM1, RPLP0, and TFRC) in 30 pairs of cancer tissues and matched adjacent tissues are shown in **Table 1** and **Figure 1**. In cancer tissues, the Ct value range for 18S rRNA was the smallest (Ct Range = 3.96), followed by TFRC (Ct Range = 6.61). The median Ct value for 18S rRNA was 8.43, lower than those of the other candidate RGs, whose Ct values ranged from 16 to 23. In matched adjacent tissues, TFRC exhibited the smallest Ct value range (Ct Range = 6.29), while 18S rRNA showed the largest range (Ct Range = 21.75).

**Table 1:** Ct values of candidate RGs in 30 pairs of tissue samples.

Cancer tissue					Adjacent tissue				
Gene symbol	C_t_ min	C_t_ max	Ct range	Mean or median^*^	Gene symbol	C_t_ min	C_t_ max	Ct range	Mean or median^*^
β-actin	15.69	32.65	16.96	18.91	β-actin	26.17	37.00	10.83	31.93
18S rRNA	6.98	10.94	3.96	8.19	18S rRNA	12.43	34.18	21.75	15.74
PUM1	13.98	27.88	13.90	23.21	PUM1	24.40	35.61	11.21	32.16
RPLP0	12.78	25.47	12.69	15.51	RPLP0	23.56	34.97	11.41	29.85
TFRC	19.65	26.26	6.61	22.19	TFRC	27.85	34.14	6.29	30.91

*Mean Ct values were provided when the data exhibited normal distribution. Otherwise, the median values were provided.

**Figure 2:**
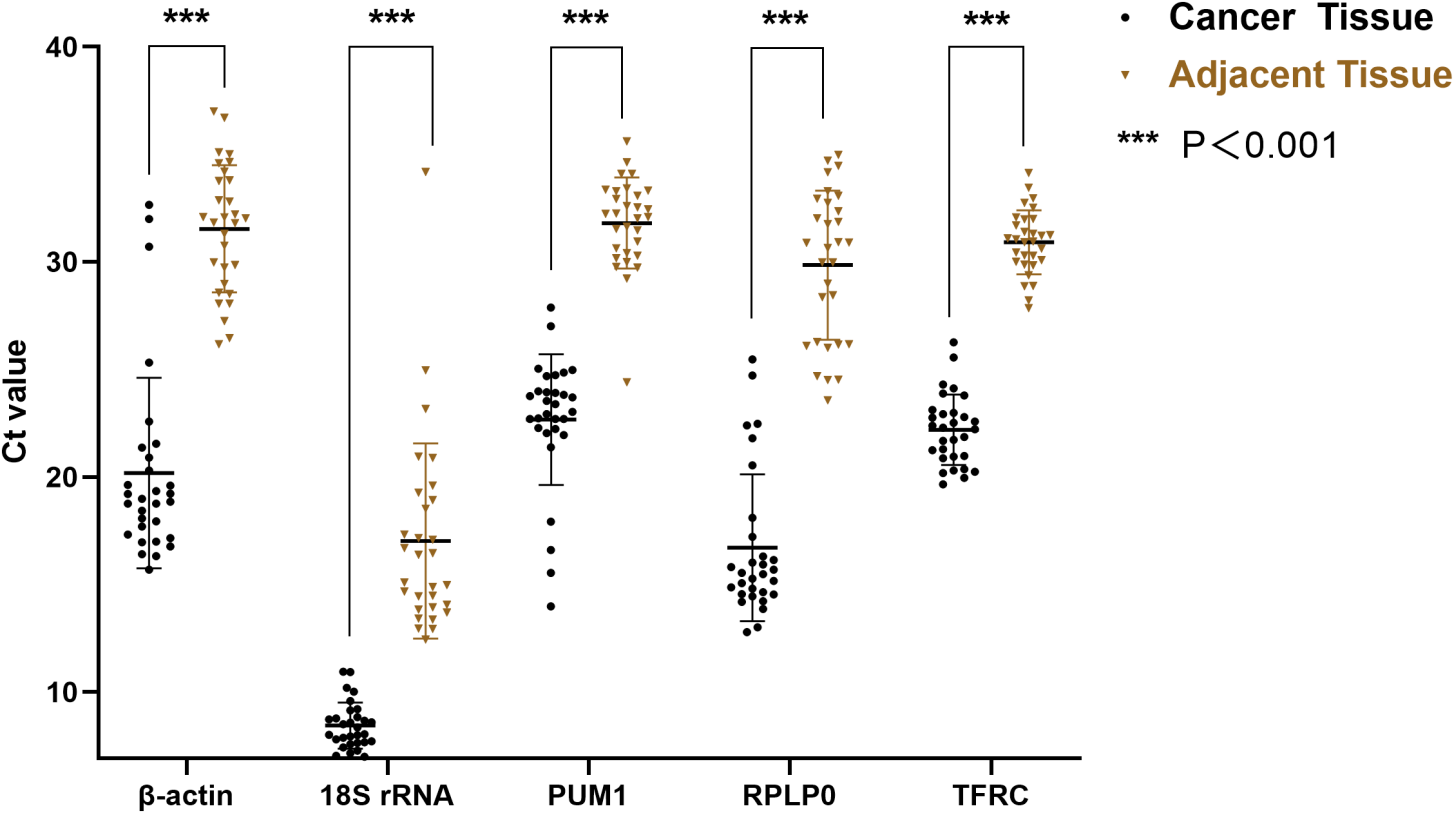
Comparison of Ct values between cancer tissues and matched adjacent tissues. The Ct values in cancer tissues were significantly higher than those in adjacent tissues. T-tests were used for β-actin and TFRC, while non-parametric tests were applied to 18S rRNA, PUM1, and RPLP0. *** indicates P < 0.001.

The differences in candidate RGs between cancer tissues and matched adjacent tissues were analyzed using t-tests or non-parametric tests. Ct values of each candidate RG in cancer tissues were significantly higher than those in matched adjacent tissues (**Figure 2**).

The samples were further classified into subgroups based on clinical and histopathological features (**Supplementary Figures S3**-**S7**). After subgroup classification by age, grade, tumor diameter, metastasis, ER, PR, Ki67, molecular subtype, and TNM stage, the Ct values of adjacent tissues remained higher than those of cancer tissues (**Supplementary Figures S3**-**S5**). In cancer tissues, none of the candidate RGs showed an association with any clinical or histopathological features (**Supplementary Figure S6**). In adjacent tissues, the Ct value of β-actin was higher in patients younger than 50 years compared to older patients. Additionally, the Ct values of 18S rRNA and PUM1 were lower in patients with Ki67 expression ≤30% compared to those with Ki67 expression >30% (**Supplementary Figure S7**).

### 3.2 Stability analysis of candidate reference genes by GeNorm

**Figure 3:**
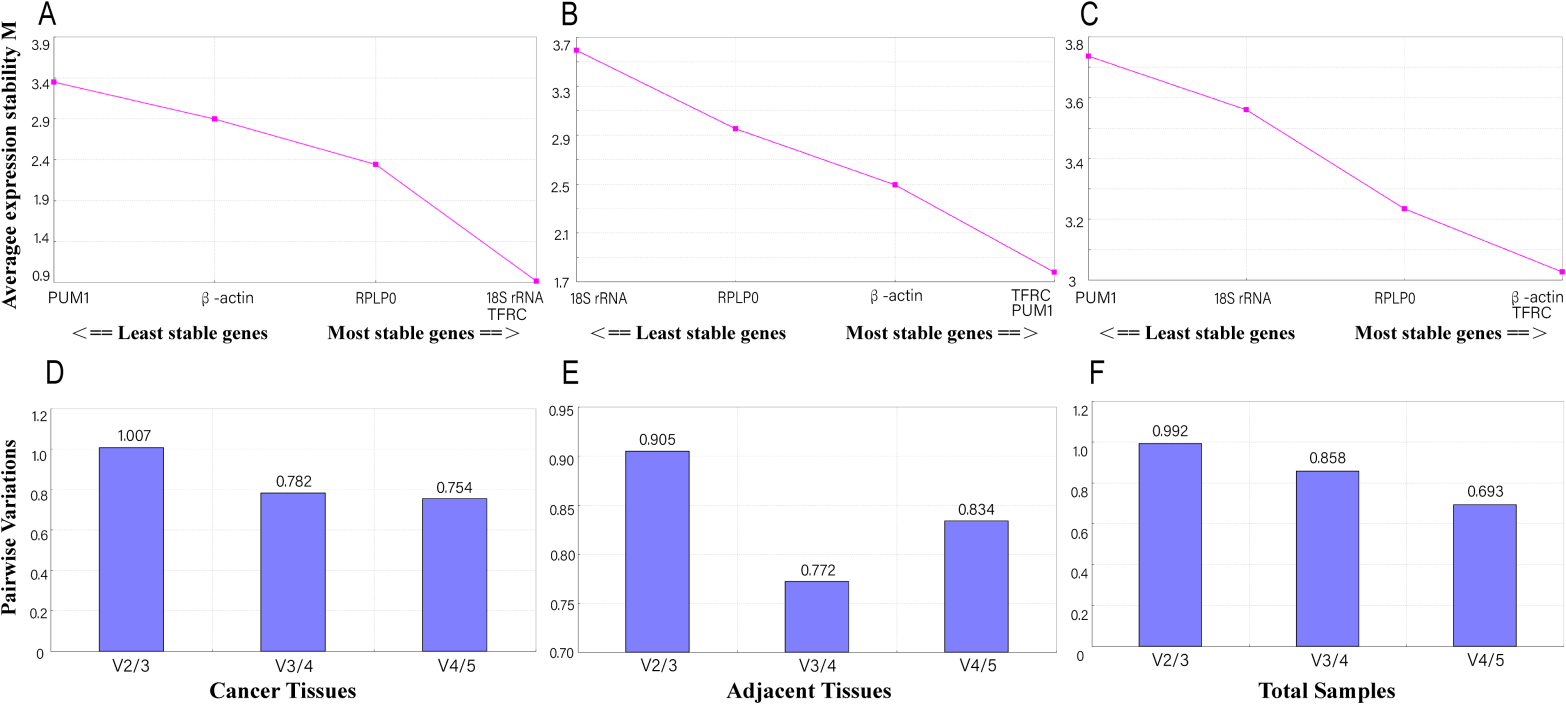
GeNorm analysis of candidate RGs. Genes with lower average expression stability (M values) indicate greater stability. **(A-C)** GeNorm analysis of M values in cancer tissues **(A)**, matched adjacent tissues **(B)**, and total samples **(C)**. The most stable candidate RGs were 18S rRNA in cancer tissues, PUM1 and TFRC in matched adjacent tissues, and β-actin and TFRC in total samples. **(D-F)** V values from GeNorm analysis in cancer tissues **(D)**, matched adjacent tissues **(E)**, and total samples **(F)**.

GeNorm calculates the average pairwise expression ratio to assess the stability of gene expression, with genes having lower M values being more stable. In cancer tissues, only the M values of 18S rRNA and TFRC (both M = 0.912) were less than 1.5 (**Figure 3A**). In contrast, all M values of the five candidate RGs were greater than 1.5 in adjacent tissues (**Figure 3B**) and in the total samples (combined cancer tissues and adjacent tissues) (**Figure 3C**). GeNorm can determine the optimal number of RGs based on a V value of less than 0.15. For the five candidate RGs, the V values were all greater than 0.15 in cancer tissues (**Figure 3D**), adjacent tissues (**Figure 3E**), and total samples (**Figure 3F**).

### 3.3 Stability analysis of candidate reference genes by NormFinder

Five candidate RGs were ranked based on their stability values (SV) calculated by NormFinder. Genes with higher SV values were considered less stable. The most stable candidate RGs were 18S rRNA and TFRC in cancer tissues (both SV = 0.456), TFRC in matched adjacent tissues (SV = 1.638), and TFRC in total samples (SV = 1.288). The least stable RGs were PUM1 in cancer tissues, 18S rRNA in adjacent tissues, and RPLP0 in total samples (**Table 2**).

**Table 2:** Expression stability ranking of the five candidate reference genes based on five algorithms.

Cancer tissues											
Reference gene	GeNorm		NormFinder		BestKeeper			Δ Ct		RefFinder	
	M	Rank	SV	Rank	SD	CV	Rank	Avg.Ct	Rank	GM	Rank
β-actin	2.899	4	3.068	3	3.05	15.11	5	3.81	4	5.00	5
18S rRNA	0.912	1	0.456	1	0.84	9.93	1	2.58	2	1.41	2
PUM1	3.347	5	3.641	5	2.00	8.85	3	4.02	5	3.46	3
RPLP0	2.341	3	3.136	4	2.61	15.61	4	3.76	3	3.46	3
TFRC	0.912	1	0.456	1	1.29	5.83	2	2.56	1	1.19	1
Adjacent tissues											
Reference gene	GeNorm		NormFinder		BestKeeper			Δ Ct		RefFinder	
	M	Rank	SV	Rank	SD	CV	Rank	Avg.Ct	Rank	GM	Rank
β-actin	2.492	3	2.375	3	2.42	7.69	3	3.47	3	3.00	3
18S rRNA	3.596	5	4.041	5	3.18	18.70	5	4.56	5	4.73	5
PUM1	1.779	1	1.907	2	1.55	4.88	2	3.23	2	1.68	2
RPLP0	2.953	4	2.497	4	2.96	9.91	4	3.63	4	4.23	4
TFRC	1.779	1	1.638	1	1.15	3.71	1	3.10	1	1.00	1
Total samples											
Reference gene	GeNorm		NormFinder		BestKeeper			Δ Ct		RefFinder	
	M	Rank	SV	Rank	SD	CV	Rank	Avg.Ct	Rank	GM	Rank
β-actin	3.555	4	2.763	2	6.27	24.26	4	3.85	3	3.13	4
18S rRNA	3.101	3	2.938	4	4.30	33.83	1	3.95	4	2.63	3
PUM1	2.364	1	2.887	3	4.69	17.33	3	3.83	2	2.06	2
RPLP0	3.803	5	3.356	5	6.70	28.77	5	4.17	5	5.00	5
TFRC	2.364	1	1.288	1	4.36	16.43	2	3.21	1	1.19	1

M, Expression stability value; SV, Stability value; SD, Standard deviation; CV, Coefficient of variation; Avg.Ct, Average ΔCt; GM, Geometric mean.

### 3.4 Stability analysis of candidate reference genes by BestKeeper

BestKeeper evaluated the stability of gene expression based on the standard deviation (SD) and coefficient of variation (CV). SD values greater than 1 suggest unstable gene expression. 18S rRNA was the most stable gene in cancer tissues (SD = 0.84) and total samples (SD = 4.30). TFRC was the most stable gene in adjacent tissues (SD = 1.15). Only the SD value of 18S rRNA in cancer tissues was lower than 1 (**Table 2**).

### 3.5 Stability analysis of candidate reference genes by ΔCt

TFRC exhibited the lowest average Ct (Avg.Ct) in cancer tissues, matched adjacent tissues, and total samples. The highest Avg.Ct values were observed for PUM1 in cancer tissues, 18S rRNA in adjacent tissues, and RPLP0 in total samples (**Table 2**).

### 3.6 Stability analysis of candidate reference genes by RefFinder

RefFinder calculated the geometric mean (GM) value for comprehensive ranking. The gene with the lowest GM value is considered the most stable. In cancer tissues, TFRC had the best stability (GM = 1.19), followed by 18S rRNA (GM = 1.41). TFRC was also the most stable gene in matched adjacent tissues (GM = 1.00) and total samples (GM = 1.19). The least stable candidate RGs were β-actin in cancer tissues, 18S rRNA in adjacent tissues, and RPLP0 in total samples (**Table 2**).

## 4 Discussion

This study evaluated the stability of the five candidate RGs in breast cancer tissues and matched adjacent tissues using RT-qPCR. The results indicated that these genes were not suitable for normalization when both cancer and matched adjacent tissues were analyzed together. Our study suggests that However, 18S rRNA and TFRC were found to be suitable reference genes for normalization when cancer tissues were analyzed individually, although further research is needed to confirm this.

To ensure coverage of distinct biological categories and expression levels, we selected 18S rRNA (a high-abundance rRNA), RPLP0 (a ribosomal protein), PUM1 (an RNA-binding protein), and TFRC and ACTB (commonly used mRNA reference genes). While the five candidate RGs has been shown to be stably expressed in various breast cancer cell lines, it is important to note that gene expression stability in cell lines may not always reflect that in tissue samples. Previous studies have reported that reference gene stability can differ significantly between cell lines and tissue types (27–29). In this study, 18S rRNA and TFRC were identified as potential RGs for breast cancer tissues due to lower variability and lack of association with clinicopathological parameters. 18S rRNA has previously been shown to be stably expressed in various breast cancer cell lines (30). In contrast, the five RGs tested were not suitable for use in adjacent tissues due to their high variability and low expression. Differential expression of β-actin in patients of different ages, and of RPLP0 and PUM1 in patients with varying Ki67 levels, further demonstrated that β-actin, RPLP0, and PUM1 were not suitable as RGs in adjacent tissues. Consistent with our findings, McNeill et al. reported that RPLP0 was differentially expressed in benign and malignant breast tumors (31). β-actin has also been shown to be differentially expressed in normal and malignant breast tissues, and may promote breast cancer cell proliferation and tumor invasiveness (10, 32–34). However, Tilli et al. suggested that PUM1 is a suitable endogenous gene for breast cancer tissue and cellular studies (35). Clinicopathological classification enhances the clinical application of cancer research. To the best of our knowledge, our study is the first to integrate clinicopathological parameters in the selection of RGs for breast cancer research.

Currently, there is no universal standard for evaluating RGs. Widely recognized methods for RG evaluation include GeNorm, NormFinder, BestKeeper, and ΔCt. Thresholds for GeNorm (M value < 1.5) and BestKeeper (SD < 1) are commonly used to screen for stable RGs. In our study, 18S rRNA was the only gene in cancer tissues that met both thresholds (M value = 0.912, SD = 0.84). To minimize differences and select the most suitable RG, we also used RefFinder. RefFinder has been widely employed for the comprehensive evaluation of RGs in both normal and disease states (36, 37). Our results indicated that TFRC ranked first in cancer tissues, followed by 18S rRNA. Therefore, 18S rRNA and TFRC could be used as RGs in breast cancer tissues. Similarly, Majidzadeh et al. concluded that TFRC is a suitable RG for detecting urokinase plasminogen activator in breast cancer (38), and 18S rRNA has been proposed as a potential RG for gastric cancer tissues and cell lines (39).

Some studies have reported that the expression of RGs varies across tissues, cell types, and disease stages (40, 41). For example, 18S rRNA has been used as an RG in research involving the MCF-7 breast cancer cell line (42), while it was the least stable RG in papillary thyroid cancer (43). PUM1 was identified as an appropriate RG in renal and colon cancers, but was the least stable in gastric cancer according to TCGA data analysis (44). These inconsistencies may be attributed to differences in tissue types, sample preparation methods, and experimental conditions.

However, our study is limited by the small sample size and the number of candidate genes tested. The findings of this study should be validated with larger patient cohorts. Future studies could leverage integrative multi-omics analyses and pan-cancer immune profiling to expand and validate candidate reference genes, thereby improving their generalizability and stability across different tissue types, molecular subtypes, and immune microenvironment conditions (45, 46). With advancements in biotechnology, RNA sequencing (RNA-seq) is gradually replacing microarray technology as the preferred method for analyzing gene expression. Combining RNA-seq with RT-qPCR could provide a more accurate approach for identifying RGs (47–50). Furthermore, the use of multiple RGs for data normalization is becoming a key focus of current research (34, 51).

## 5 Conclusion

Five RGs (β-actin, 18S rRNA, PUM1, RPLP0, and TFRC) were analyzed in both breast cancer and matched adjacent tissues. Our study identified that TFRC and 18S rRNA exhibited potential as reliable RGs in breast cancer tissues. However, the five genes were not suitable as RGs in matched adjacent tissues, either alone or when combined with breast cancer tissues. Further research is needed to identify more appropriate RGs for breast cancer.

## Data Availability

The original contributions presented in the study are included in the article/**Supplementary Material**. Further inquiries can be directed to the corresponding author.

## Supplementary material

Supplementary Table S1 Clinical and histological features of patients in the study.

Supplementary Figure S1 The expression levels (Ct values) of five candidate reference genes (β-actin, 18S rRNA, PUM1, RPLP0, and TFRC) in four breast cancer cell lines (MDA-MB-231, BT-474, MCF-7, and MDA-MB-468).

Supplementary Figure S2 **(A-E)** Melt curve of five candidate reference genes (β-actin, 18S rRNA, PUM1, RPLP0, and TFRC) using RT-qPCR. **(F)** Agarose gel electrophoresis of PCR products for the five genes.

Supplementary Figure S3 The Ct values of cancer and matched adjacent tissues in patients stratified by age, grade, tumor sizeand metastasis.

Supplementary Figure S4 The Ct values of cancer and matched adjacent tissue in patients stratified by ER, PR,HER2 and Ki67.

Supplementary Figure S5 The Ct values of cancer and matched adjacent tissues in patients stratified by subtype and clinical TNM stage.

Supplementary Figure S6 Comparison of Ct values of cancer tissues in subgroups with different clinicopathological parameters.

Supplementary Figure S7 Comparison of Ct values of matched adjacent tissuesin subgroups with different clinicopathological parameters.
