# Supplementary material for: Identification of endogenous reference genes for RT-qPCR analysis in breast cancer and matched adjacent tissues
Source: Front Oncol. 2026 Jan 20;15:1702210. doi: 10.3389/fonc.2025.1702210 (PMC12864094; doi:10.3389/fonc.2025.1702210)
Supplement: Supplementary Table S1 — Clinical and histological features of patients in the study. [file Table1.docx]

****Supplementary Table S1**** Clinical and histological features of patients in the study

| **Clinical and histological parameters** |  | **Cases** |
| --- | --- | --- |
| Age | ≤50 | 11 |
|  | ＞50 | 19 |
| Grade | Ⅰ | 2 |
|  | Ⅱ | 17 |
|  | Ⅲ | 11 |
| Size (mm) | ≤20 | 12 |
|  | ＞20 | 18 |
| Lymph Node Metastasis | No | 16 |
|  | Yes | 14 |
| ER | Negative | 11 |
|  | Positive | 19 |
| PR | Negative | 9 |
|  | Positive | 21 |
| HER2 | Negative | 20 |
|  | Positive | 10 |
| Ki-67 Expression | Low (≤ 30%) | 20 |
|  | High (＞ 30%) | 10 |
| Molecular Subtype | Luminal | 18 |
|  | HER2-positive | 9 |
|  | TNBC | 3 |
| TNM Stage | Ⅰ | 6 |
|  | Ⅱ | 17 |
|  | Ⅲ | 7 |
