# Supplementary figures and images for: Identification of endogenous reference genes for RT-qPCR analysis in breast cancer and matched adjacent tissues
Source: Front Oncol. 2026 Jan 20;15:1702210. doi: 10.3389/fonc.2025.1702210 (PMC12864094; doi:10.3389/fonc.2025.1702210)

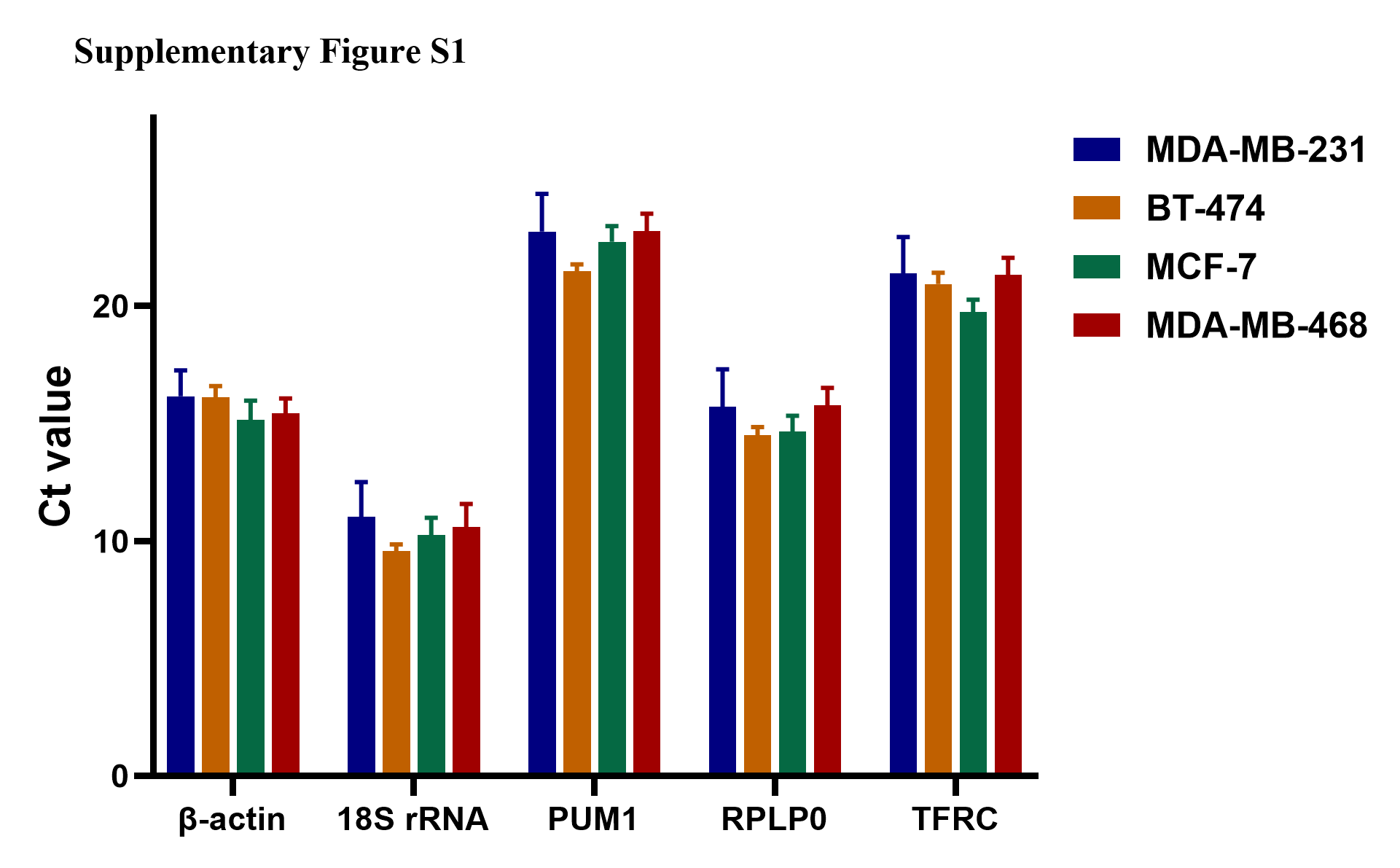

Supplement: Supplementary Figure S1 — The expression levels (Ct values) of five candidate reference genes (β-actin, 18S rRNA, PUM1, RPLP0, and TFRC) in four breast cancer cell lines (MDA-MB-231, BT-474, MCF-7, and MDA-MB-468). [file Image1.tif]

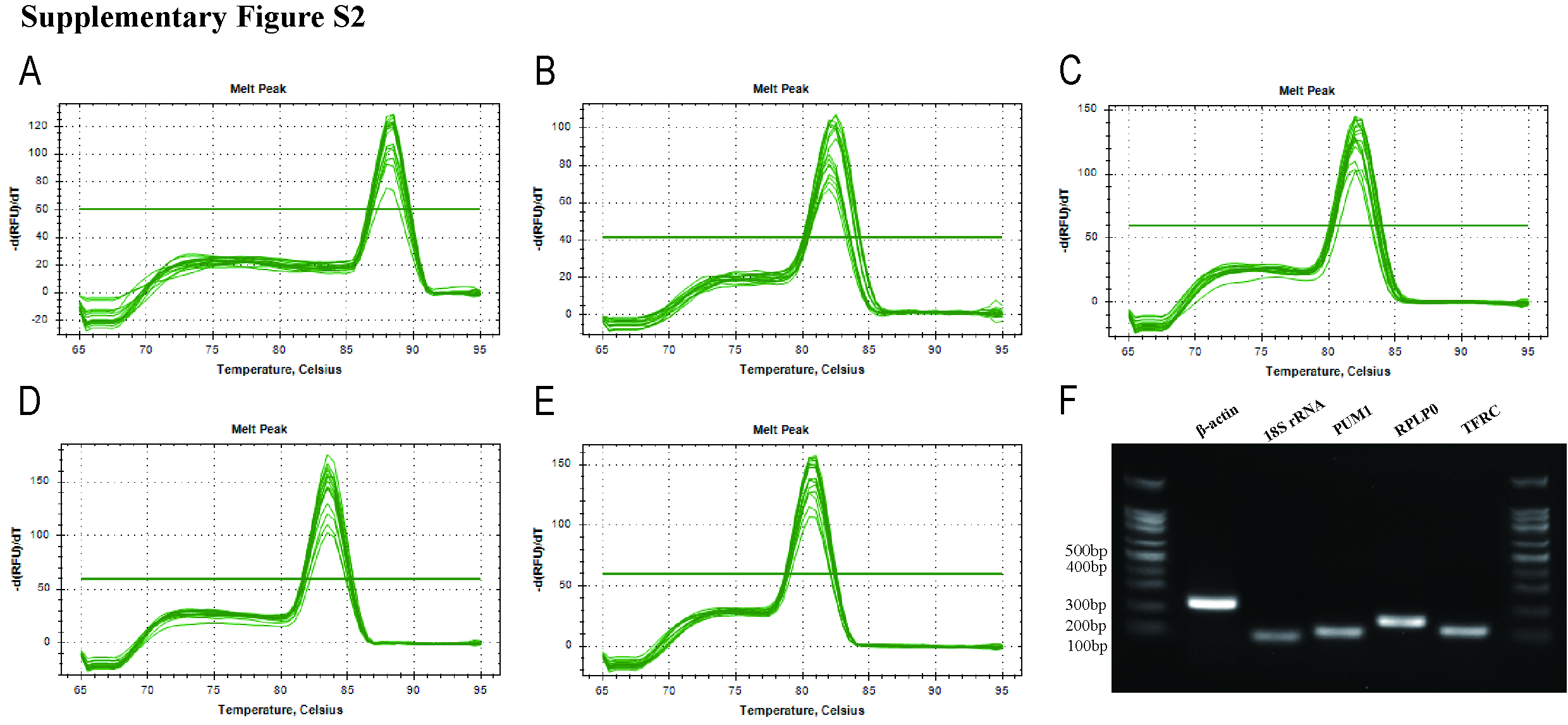

Supplement: Supplementary Figure S2 — (A-E) Melt curve of five candidate reference genes (β-actin, 18S rRNA, PUM1, RPLP0, and TFRC) using RT-qPCR. (F) Agarose gel electrophoresis of PCR products for the five genes. [file Image2.tif]

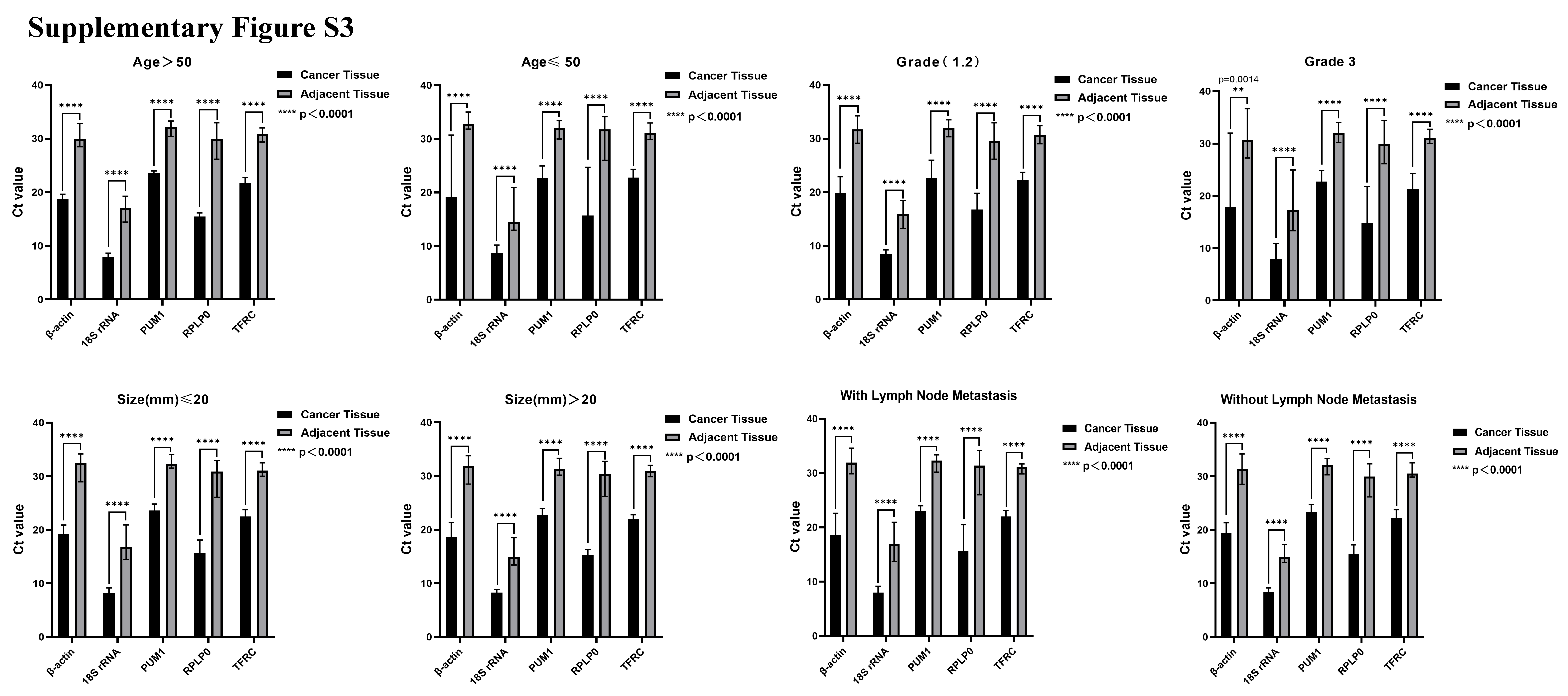

Supplement: Supplementary Figure S3 — The Ct values of cancer and matched adjacent tissues in patients stratified by age, grade, tumor sizeand metastasis. [file Image3.tif]

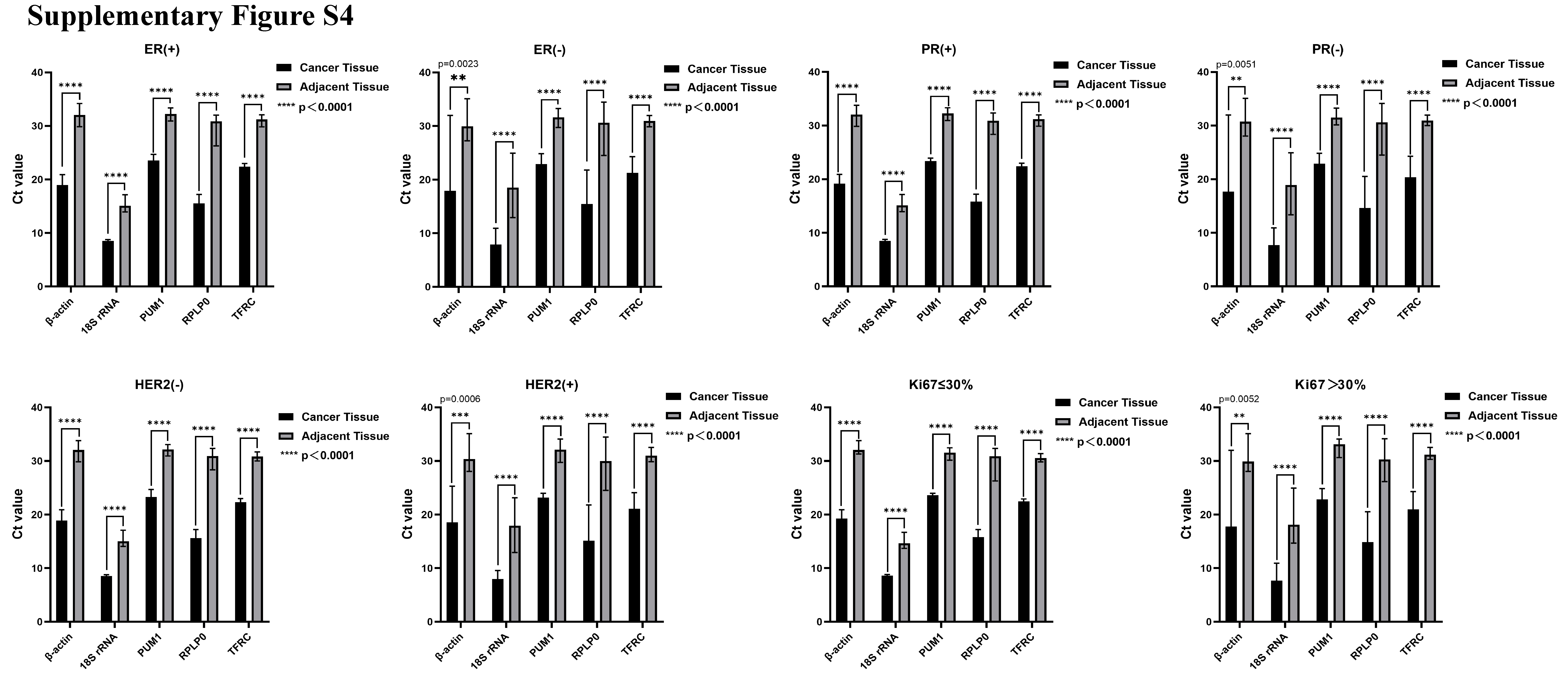

Supplement: Supplementary Figure S4 — The Ct values of cancer and matched adjacent tissue in patients stratified by ER, PR,HER2 and Ki67. [file Image4.tif]

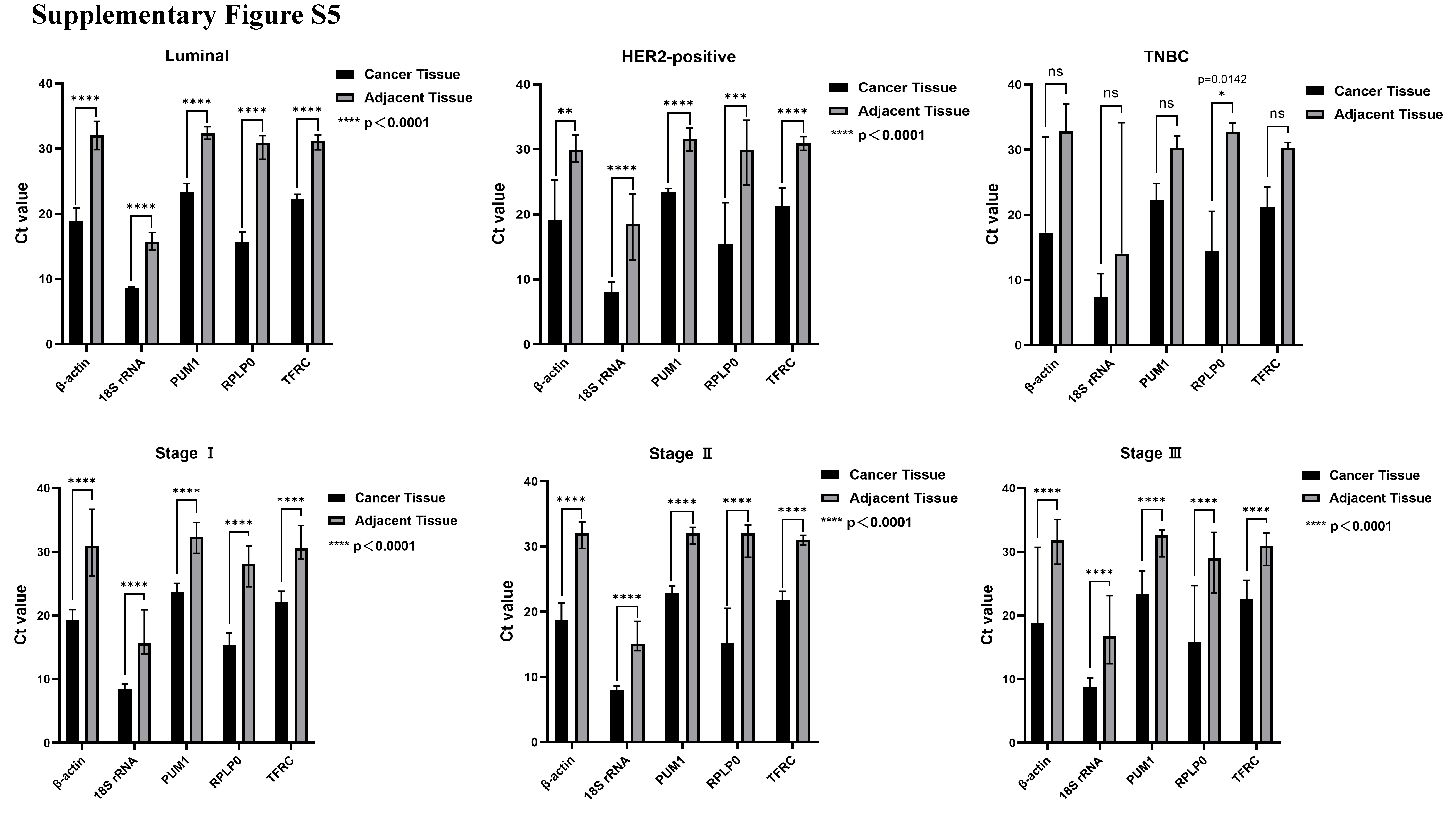

Supplement: Supplementary Figure S5 — The Ct values of cancer and matched adjacent tissues in patients stratified by subtype and clinical TNM stage. [file Image5.tif]

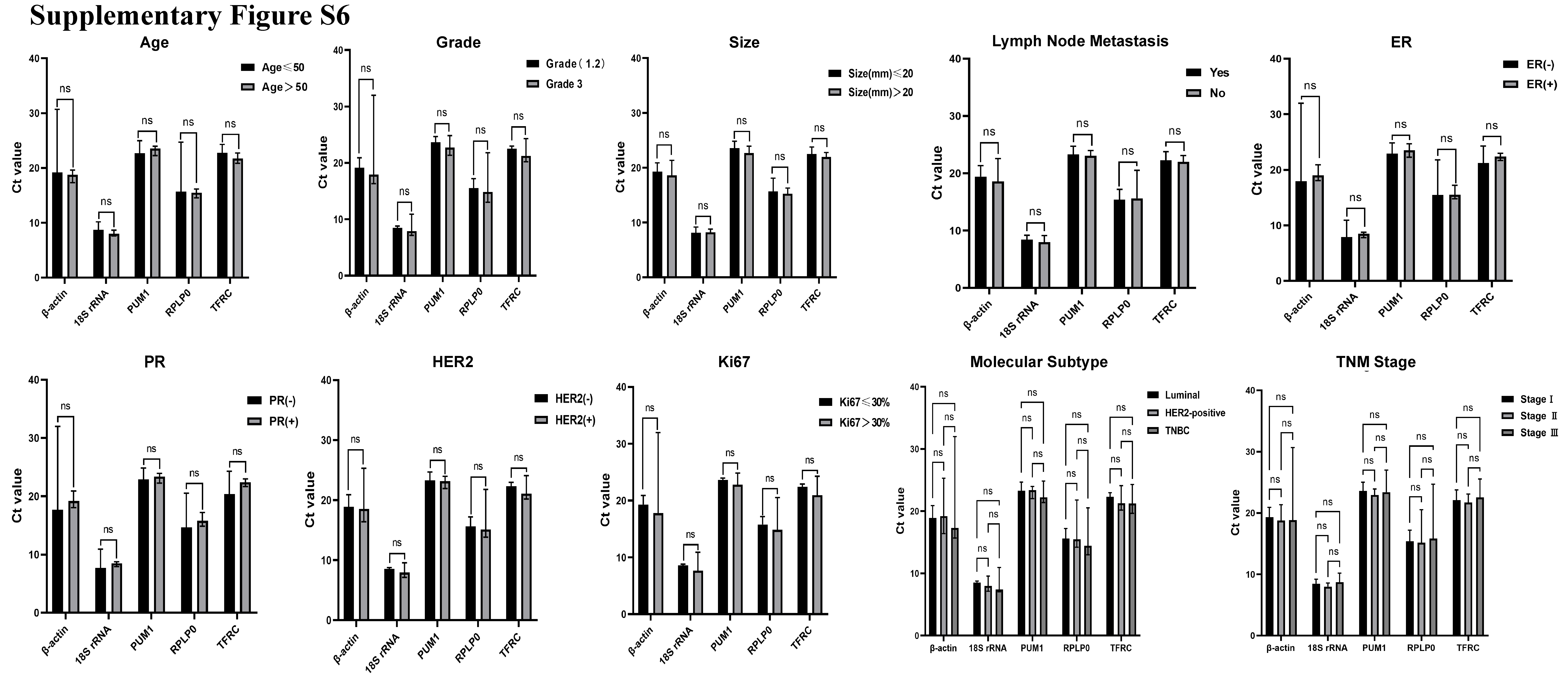

Supplement: Supplementary Figure S6 — Comparison of Ct values of cancer tissues in subgroups with different clinicopathological parameters. [file Image6.tif]

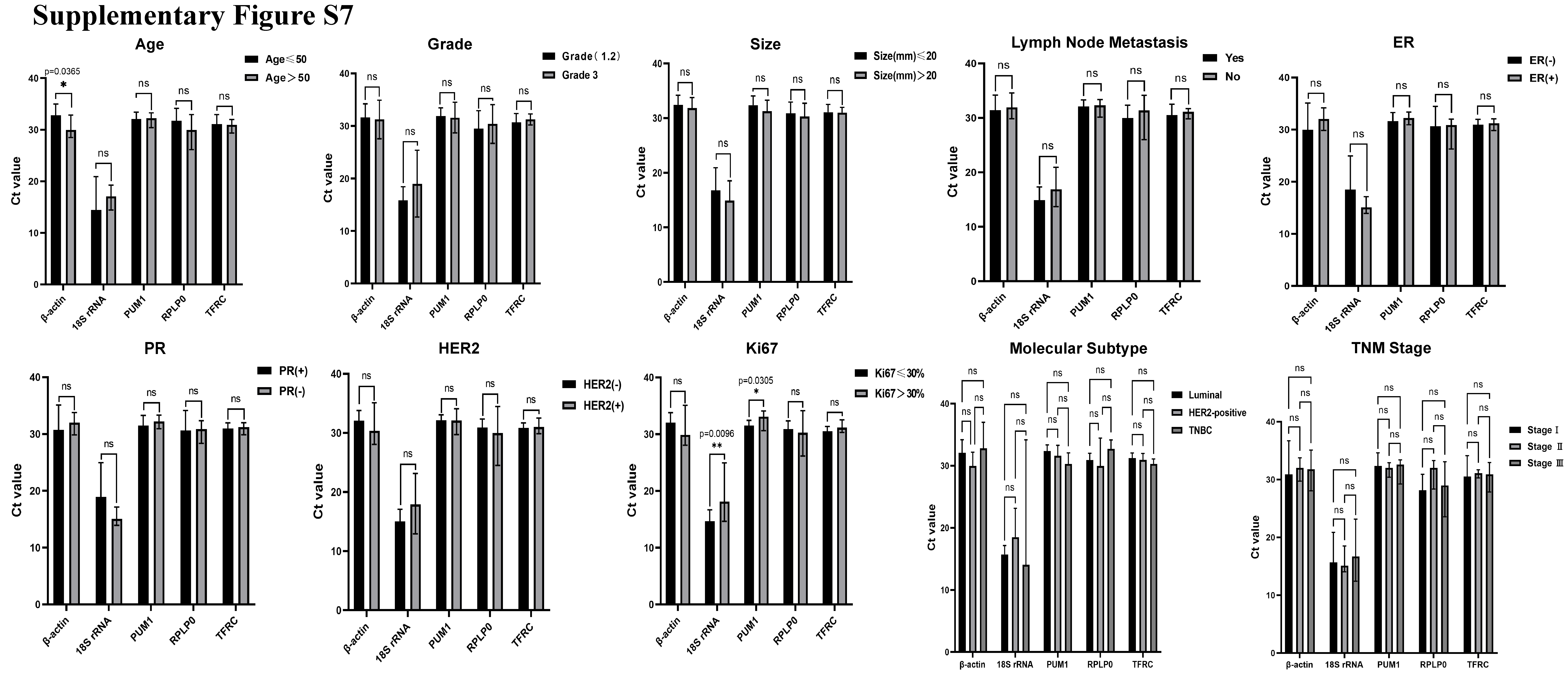

Supplement: Supplementary Figure S7 — Comparison of Ct values of matched adjacent tissuesin subgroups with different clinicopathological parameters. [file Image7.tif]
